# Stability and conductivity of self assembled wires in a transverse electric field

**DOI:** 10.1038/srep15044

**Published:** 2015-10-14

**Authors:** C. Stephenson, A. Hubler

**Affiliations:** 1 Department of Physics, University of Illinois at Urbana Champaign, Urbana, Illinois

## Abstract

Self assembling wire networks typically evolve to minimize the resistance across electrical contacts which are frequently used in a manner comparable to Hebbian learning. In this work, we demonstrate that electrical fields can also be used to cause an increase in the resistance of the wire network. We show that if such a wire is exposed to a transverse electric field, the wire is deformed in a way that depends on it’s tensile strength. We measure the wire resistance as a function of transverse field for several field strengths and show that by deforming the wire, the amplitude of the resulting shape can be modified in a controllable fashion. At a critical value of the transverse field, we show that the wire loses stability. At this point we observe thresholding behavior in that the resistance increases abruptly to a maximum value and the wire is destroyed. This thresholding behavior suggests that self assembled wires may be manipulated via an transverse electric field and demonstrates that a mechanism exists for the destruction of undesirable connections.

Self assembling particle wire networks have been studied recently as a potential means of producing complex electrical devices^1^,^2^,^3^. These systems consist of electrically polarizable particles which self assemble in response to an electric field^4^, often via dielectrophoresis^5^,^6^. However the particle-particle interactions also appear to be important^7^,^8^ and interesting instabilities are known to arise in similar systems^9^. Of particular interest is the potential for using this type of system for the growth of dynamically evolving reconfigurable structures^10^,^11^ or physically evolving networks^12^ and shape memories^13^. Self assembled wire networks have the advantageous property that the network resistance can change in response to externally imposed boundary conditions^14^, and in some cases this leads to the optimization of conductivity between nodes^15^. The dependence of conductivity on past voltage states is similar to the behavior of memristive systems^16^, and in fact memristors have also been investigated as a means of created biologically inspired adaptive systems^17^,^18^ although such devices must be constructed rather than grown.

The optimization in self-assembling wire networks can be viewed as the network learning the nature of its environment, analogous to Hebb’s learning rule^19^. However, if the network evolves only to maximize its conductivity, it is limited in its capabilities. In regular Hebbian learning, the connection weights increase monotonically over time, just as the conductivity increases monotonically to infinity in the model considered in reference^15^. In order to overcome this, a self assembled wire network must be able to increase the resistance of a connection naturally, or even destroy connections which are undesirable. In neural networks, this corresponds to having excitatory connections (which increase neuron activity) and inhibitory connections (which decrease activity). We aim to demonstrate that this can indeed happen in self assembling wires when the total electric field in the vicinity of the wire is not parallel to the wire. This effect explots an electric field dependent instability.

To do this, we first expose a self assembled wire to a small electric field perpendicular to the wire, and observe the change in wire shape and resistance. We then explore the behavior of the wire in the presence of a large perpendicular electric field. It is in this regime in which the wire begins to break down and we find thresholding behavior. Then we discuss a theory that quantitatively describes the charge transport properties as well as the observed deformation and breaking of these wires which explains the behavior of a self-assembled wire subject to a transverse electric field. The resulting behavior of the device somewhat resembles that of a memristor in that the resistance is dependent on the past voltages across the wire^16^. However, our device requires control of a parallel voltage as well as a transverse voltage and is therefore not a memristor but rather a type of voltage controlled resistor.

## Results

**Figure 1:**
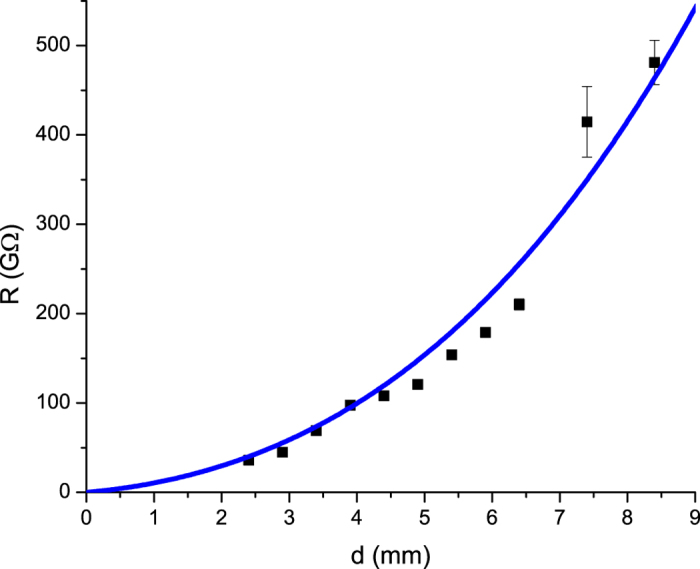
Resistance of a gap with one particle as a function of gap size. The blue line shows a best fit to Eq. 9 for the constant *A* = 0.379 *mm*^3^/*G*Ω.

Figure 1 shows the resistance of a gap of various sizes containing a single shuttling particle. This resistance was measured at a primary electrode voltage of *V* = 18 *kV*. We see that this resistance is increases nonlinearly with the distance between electrodes due to the charge carried by the shutting particle.

**Figure 2:**
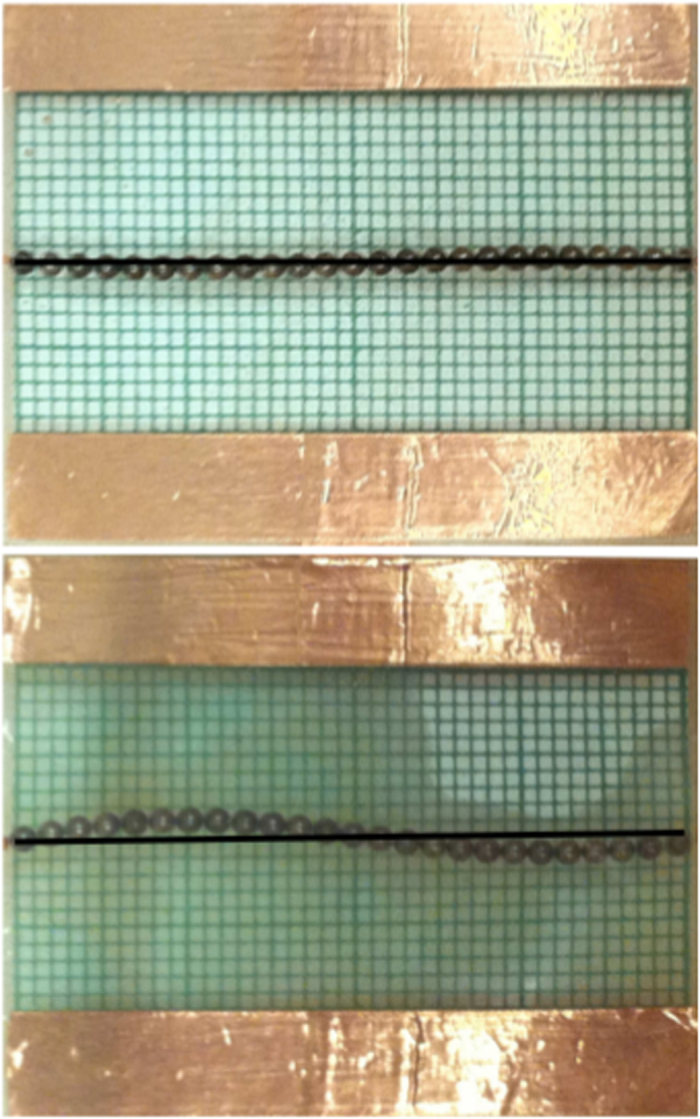
Wire formed at Δ*V* = 25 *kV* showing a typical wire at zero transverse field and the deformed wire after the transverse field is switched on.

**Figure 3:**
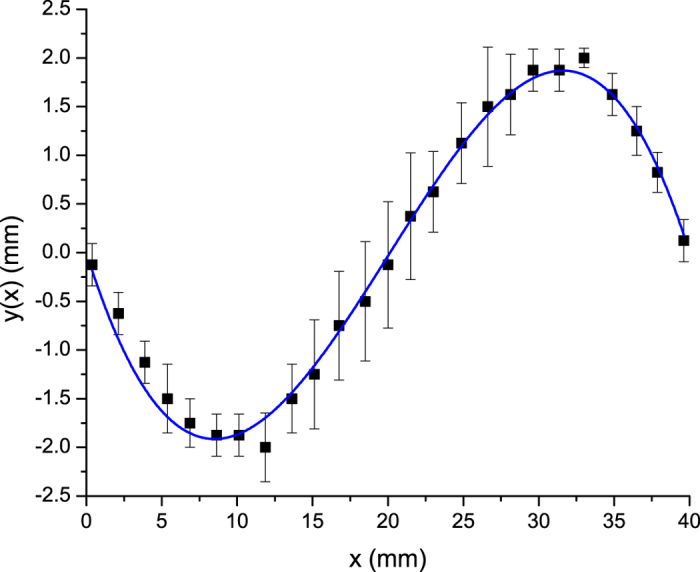
Displacement of a 25 particle wire when exposed to a *V*_^_ = 2 *kV* transverse voltage with an Δ*V* = 18 *kV* primary voltage compared to the theoretical curve for the best-fit value of *c*/*T* = 9.1 * 10^−5^ *F*/*N* as defined in Eq. 15.

In the absence of a transverse electric field, the particles form a wire connecting the two primary electrodes which is straight when averaged over multiple instances. However, when the transverse voltage is applied we observe a distortion of the wire. This behavior can be seen in Fig. 2. Figure 3 shows the shape that the normally straight wire acquires when the voltage on the secondary electrodes is nonzero but still small compared to the primary electrode voltage. The data points shown represent an average over five experimental runs with the same setup, and the error bars show the standard deviation for each particle’s displacement.

**Figure 4:**
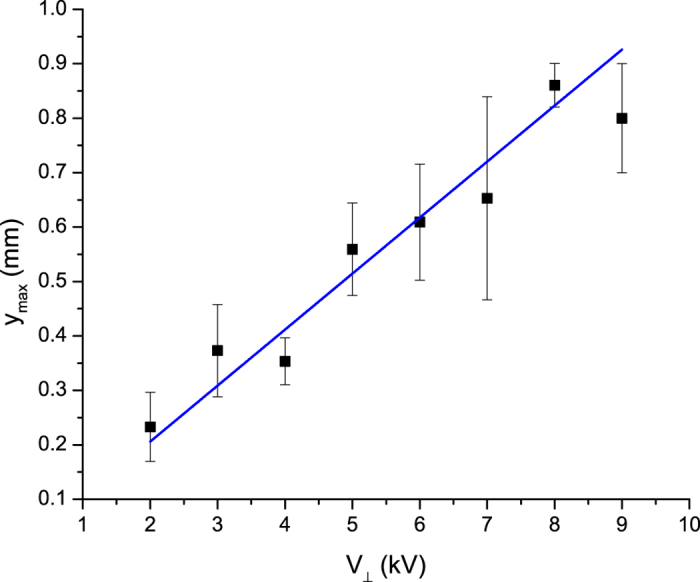
Amplitude of wire displacement of a 25 particle wire as a function of transverse voltage with a Δ*V* = 25 *kV* primary voltage compared to the the theoretical curve as defined in equation 16 for the best fit value of *c*/*T* = 7.1 * 10^−6^ *F*/*N*.

We find that the amplitude of the distortion increases linearly with the transverse voltage. Figure 4 shows the dependence of the maximum wire displacement on the transverse voltage. We see that the displacement amplitude increases linearly with the transverse voltage before the critical voltage at which the wire breaks. This behavior is explained by the same theory that predicts the shape attained by the wire.

**Figure 5:**
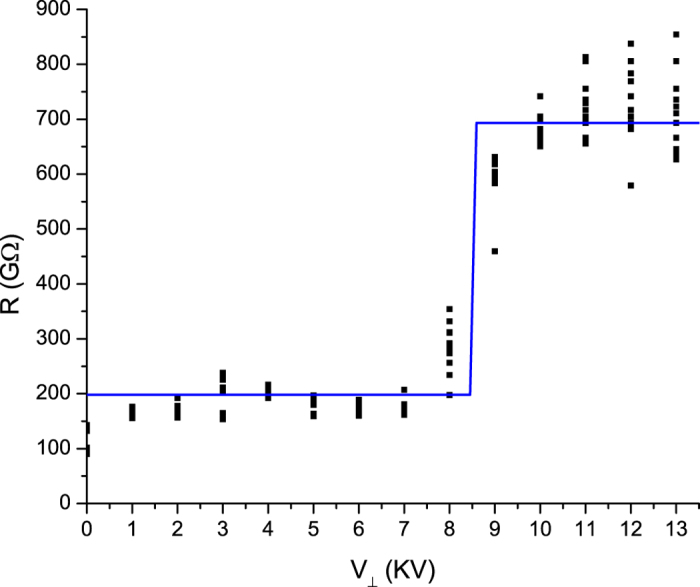
Resistance vs. transverse voltage showing a transition at *V*_*c*_ = 8.5 *kV* +/− 0.7 kV. The blue line is a best fit step-function with a low resistance of *R* = 198 *G*Ω and a high resistance of *R* = 693 *G*Ω with the transition at *V*_*c*_ = 8.5 *kV*.

Figure 5 shows the change in wire resistance as a function of the secondary electrode voltage for ten wires that were measured. We see that the wire has a resistance of *R* = 250 *G*Ω for all transverse voltages up to *V*_^_ = 13 *kV*, and transitions abruptly to a resistance state of *R* = 750 *G*Ω between *V*_^_ = 7 *kV* and *V*_^_ = 10 *kV* when the wire is destroyed, after which the resistance is constant for increasing transverse voltages. Each data point is a measurement of the average resistance of the wire over five minutes. The critical voltage was defined as the transverse voltage at which the resistance more than doubled the value measured at the previous transverse voltage. We measured a critical *V*_*c*_ = 8.5 *kV* +/− 0.7 *kV*.

## Discussion

We have observed that the particles in the wire shuttle charge along the wire in the manner of a bucket brigade^20^. The charge shuttling mechanism^21^ occurs when the conductive particle contacts one of the neighboring particles or electrodes and acquires a monopole charge. The charged particle is then attracted to the opposite electrode or an oppositely charged neighbor where it discharges and the process repeats. The net result is that charge is carried across gaps between electrodes at a rate that depends on the frequency that the shuttling particle crosses the gap. We will use this observation as well as a few simplifying assumptions to form a model of the charge distribution along the wire as well as the response of the wire to a perpendicular electric field.

**Figure 6:**
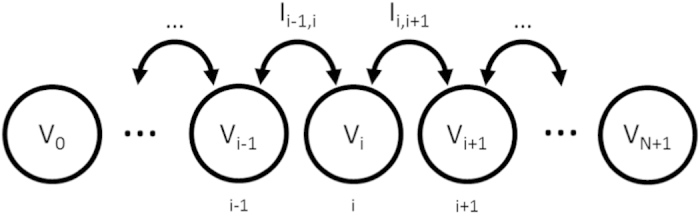
Particle chain.

We consider a chain of *N* particles each carrying a voltage *V*_*i*_ with *i* = 1, 2..., *N* as diagrammed in Fig. 6. At each end of the chain we have a particle that acts as electrode with voltages *V*_0_ and *V*_*N*+1_ respectively. We will consider the case of one positive electrode and one negative electrode such that *V*_0_ = −*V*_*N*+1_ = Δ*V*/2.

The current *I*_*i*,*j*_ is the current that flows from particle *i* to particle *j*. Since current only flows between nearest neighbors in the chain, conservation of charge then gives the equation of motion for the charge *Q*_*i*_ on particle *i* as


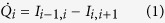


Now we introduce the (possibly non-ohmic) conductivity *σ*_*i*,*j*_ between the nearest neighbor particles *i* and *j* and write the currents:


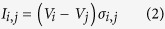


This expression is simply Ohm’s law.

Now we require that the system has reached the stationary state in which the time averaged particle charges are no longer varying. In this case, the left side of Eq. 1 is zero and we have *I*_*i*−1,*i*_ = *I*_*i*,*i*+1_ for all *i* = 1, 2..., *N*. This implies that the currents between nearest neighbor particles has also reached a constant value *I*, and so equation 2 becomes


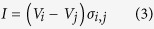


This equation can be solved for the voltage. The solution is linear, and after enforcing the boundary conditions we have


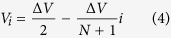


And equation 3 can be rewritten as


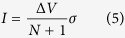


Which only depends on the electrode voltages and the total number of particles. The dependence of the conductance *σ* on the gap size may be evaluated at least approximately by considering the dynamics of a single particle shuttling between two identical neighboring particles held at an electrode voltage difference Δ*V*_*e*_. Provided the gap is large compared to the radius of the particle, the leading order term in the force on the particle is simply the monopole term^22^. Taking the overdamped approximation, the equation of motion of the particle crossing the gap is


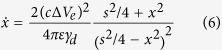


where *c* is the particle capacitance, *γ*_*d*_ is the damping coefficent due to the oil, and *s* is the separation between the center of the neighboring particles. From this we calculate the time *t* required for the particle to cross the gap once. Introducing the constant *A* = 2*c*^3^(Δ*V*_*e*_)^2^/(4*πεγ*_*d*_) and the gap size *d* = *s* − 4*r* we find


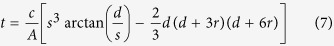


Since the particle transfers a charge *c*Δ*V*_*e*_ every time it crosses the gap, we have the conductivity as it depends on *d*


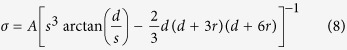


and the gap size dependent resistance


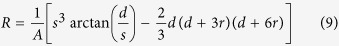


This completes the description of the wire conductivity as it depends on the idealized shuttling motion of the equally spaced particles. However, during the real bucket brigade motion of many particles, much larger gaps may appear in the wire at the expense of smaller gaps. This causes the conductivity of the wire to momentarily drop as charge cannot be carried effectively across large gaps. When this happens, the particles that are still in contact with an electrode charge up to the electrode voltage, and particles that are no longer in contact with an electrode rapidly become neutral.

We consider now the case of a single gap forming in the wire. We assume that the gap has an equal chance of occurring at any of the *N* + 1 possible sites. Once the gap has formed, the particle to the left of the gap will have the left electrode voltage, and the particle to the right will have the right electrode voltage. Since current can no longer flow across the gap in the wire, the particles on either side of the gap will acquire a charge dependent upon their capacitance *c* and the voltage they reach. Therefore, these particles will experience a force *F*_*i*_ = *cV*_*i*_* E*_^_ perpendicular to the length of the wire if exposed to a perpendicular external electric field *E*_^_. The force *F*_*i*_ averaged over all possible gaps can be found by considering the probability that the gap is on either side of the particle. If we assume that the gap is equally likely to occur at any of the sites, then there are *i* sites on one side (left) of the particle, and *N* − *i* + 1 sites on the other side (right). Therefore there is a probability of *i*/(*N* + 1) that the gap is to the left of particle *i* and a probability of (*N* − *i* + 1)/(*N* + 1) that the gap is to the right. The magnitude of the perpendicular force on the particle is the same in either case, but the direction of the force is determined by which side of the particle the gap is on. If we say the force is positive when the gap is to the left of the particle, and negative when it is to the right, we find that the expectation value of the force averaged over all possible gaps is


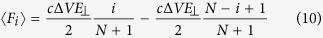


or


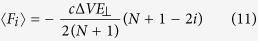


That is, the average force on the particle at site *i* is determined by the probability that the gap is to the left of site *i* and the probability that the gap is to the right of site *i*. We see that this changes linearly along the length of the wire. This force acts perpendicular to the straight wire

If the stretch of the wire is small, then the total length is not significantly different from the unstretched length and the gap size may be approximated as (*L* − 2*Nr*)/(*N* + 1). We may use this to calculate the force parallel to the direction of the wire. The force between a sphere of charge *Q* and an uncharged sphere of equal radius with a gap of size *s* between them is


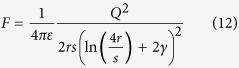


where *γ* is the Euler-Mascheroni constant and *ε* is the permittivity of the medium. This is accurate to leading order in *s*/*r*. Using the gap size in the wire for *s*, and the charge *Q* = *c*Δ*V*, we have the force parallel to the wire as


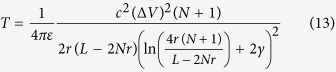


Which is the effective tension in the wire. From this, we can calculate the displacement of the wire in a transverse electric field. We will do this by taking the approximation of a continuous wire *i* → *x*(*N* + 1)/*L* so that 〈*F_*i*_*〉 → 〈*F*(*x*)〉 = −*c*Δ*VE*_^_(1/2 − *x*/*L*) where *L* is the total length of the wire. Assuming a small displacement *y*(*x*) and a viscous damping term 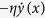 the displacement of the wire in a transverse electric field satisfies the equation of motion


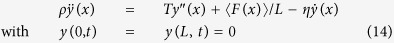


Where T is the restoring force acting as the tension in the wire from above. In the steady state, 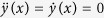 and we can solve for the displacement of the wire


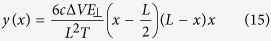


This shows that the maximum displacement *y*_*max*_ of the wire should increase linearly with the perpendicular electric field.


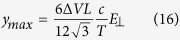


Using the tension calculated above, and writing 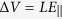 where 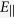 is the electric field produced by the electrodes, which is parallel to the wire we find


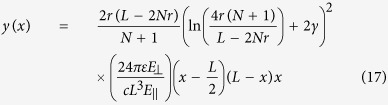


This gives the average wire displacement to leading order in the number of gaps. We can gain one further simplification by approximating the capacitance of the charged particle as the capacitance of a single sphere *c* ≈ 4*πεr* (although this approximation is not valid, it will provide a rough estimate) which gives


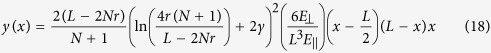


This is entirely in terms of known quantities.

We can also approximate the perpendicular electric field required to break the wire. As the wire distorts, the force due to the perpendicular field gains a component parallel to the wire that acts against the tension force. At the critical perpendicular field strength, this component becomes larger than the tension in the wire and the wire breaks. This condition can be written as


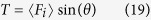


where *θ* is the angle that the wire makes with the x-axis. The lowest field at which the wire can break can be found by taking 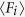 to be its maximum value of 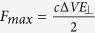 and computing the maximum value of *sin*(*θ*) as a function of the electric fields. We find the value of sin(*θ*) using the derivative of eq. 15. This gives 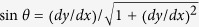. Using the maximum value for the derivative on the wire *dy*/*dx* = 24*F*_*max*_/*T* we find the relationship for the critical field to be


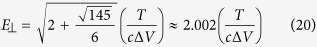


Using eq. 13 with the largest possible gap size for the tension and our approximation of the capacitance *c* ≈ 4*πεr* leads to a critical field of


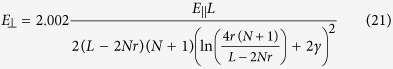


Using the parameter values from the experiment and the approximations above, we find the value of *c*/*T* = 1.2 * 10^−5^ *F*/*N* which is much lower than our measured *c*/*T* = 9.1 * 10^−5^ *F*/*N*. We also calculate a critical field strength of *E*_^_ = 334 kN/C which corresponds to a perpendicular electrode voltage of *V*_*c*_ = 6.7 kV. This is somewhat lower than the measured *V*_*c*_ = 8.5 +/− 0.7 *kV* breaking voltage. While these estimates all differ from the experimental values, some discrepancy is expected since we have used the capacitance of an isolated sphere as an approximation to the capacitance of the particles in the wire. Since the particles in the wire are interacting, their capacitances may differ somewhat from the values used in this approximation.

To summarize, we find that the conductivity of a self assembled particle wire can be related to the conductivity of a single particle charge shuttle. We can therefore view the wire as a chain of charge shuttling particles, similar to a bucket brigade. We also find that a self assembled particle wire can be distorted or even destroyed by a strong enough perpendicular electric field. This can be understood as being caused by gaps that form in the wire during the bucket brigade conduction motion of the particles which cause sections of the wire to have a nonzero time averaged charge. This effect may be exploited to use self assembled wires as a type of voltage controlled resistor, and demonstrates that self assembling wire connections are capable of increasing resistance in response to changes in the local environment.

## Methods

**Figure 7:**
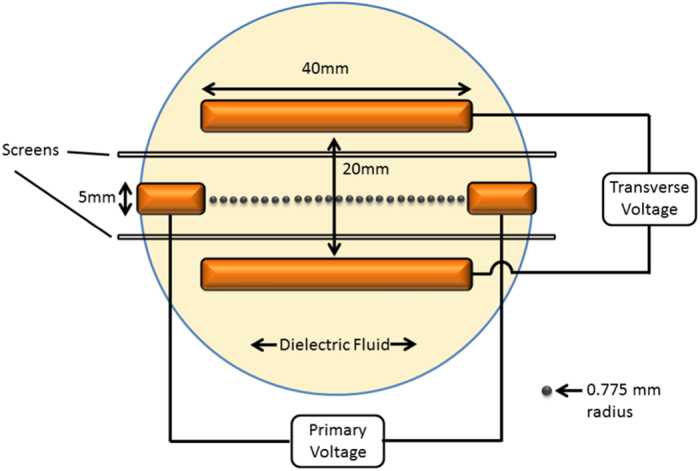
Diagram of the wire experiment.

Figure 7 shows a diagram of the experiment. The setup consists of two primary electrodes which are 5 *mm* wide and separated by a distance of *L* = 40 *mm*. The electrodes are immersed in a dielectric fluid (castor oil) and a voltage (the primary voltage) is applied across them. The oil contains *N* = 25 steel conducting spheres of radius *r* = 0.775 *mm* which self assemble into a wire. The particles in the wire transport charge by undergoing a bucket brigade motion.

Parallel to the line connecting the two primary electrodes are two additional *L* = 40 *mm* long secondary electrodes separated by a distance of *d* = 20 *mm*. A voltage (the transverse voltage) is applied to these electrodes and the wire changes its configuration, thus altering the displacement of the wire and its resistance.

Wires were allowed to equilibriate in the applied fields for five minutes, after which digital images of the wires were taken by a camera mounted above the experiment. Two examples of these images can be seen in Fig. 2. To measure the wire’s displacement, the pixel position of each particle in the image was recorded. A 1 *mm* grid below the experiment was used to determine the pixel size and so convert the pixel position into to the real position of the particle in the experimental area. Particle positions were recorded using coordinates centered at the primary positive electrode. The line connecting the primary electrode was defined to be the *x*-axis.

The wire resistance measurements were taken using an identical setup with the addition of two porous nonconducting screens by the transverse electrodes to prevent the particles from forming a connection between this electrode pair at higher transverse voltages. A 0.5 *M*Ω resistor was placed in series with the primary voltage source and the wire. The time averaged voltage across this series resistor was recorded and use to compute the current through the circuit and therefore the total resistance of the wire. The resistance of the formed wire was measured as a function of the secondary electrode voltage while the voltage across the main electrode remains fixed.

**Figure 8:**
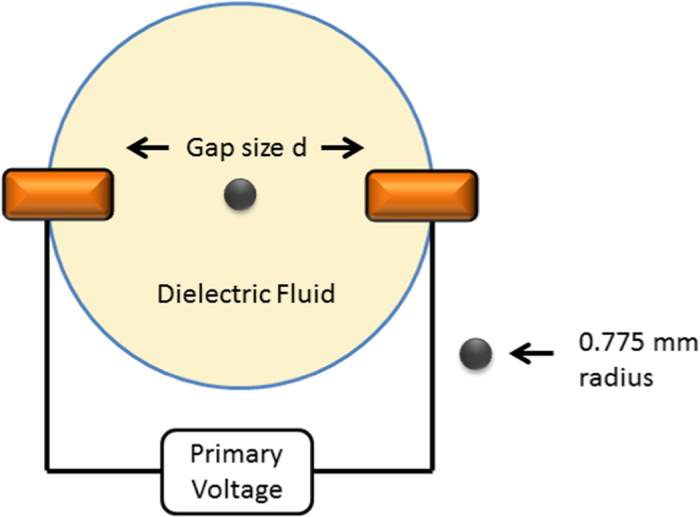
Diagram of the shuttle experiment.

Figure 8 shows a diagram of the experiment used to measure the current carried by a single shuttling particle. The gap size *d* was varied in this experiment to find the resistance as a function of gap size. A 0.5 *M*Ω resistor was placed in series with the primary voltage source and the voltage across this resistor was measured to compute the resistance of the gap with the particle in it.

## Additional Information

**How to cite this article**: Stephenson, C. and Hubler, A. Stability and conductivity of self assembled wires in a transverse electric field. *Sci. Rep.* **5**, 15044; doi: 10.1038/srep15044 (2015).
